# Intrauterine Growth Restricted Rats Exercised before and during Pregnancy: Maternal and Perinatal Repercussions

**DOI:** 10.1155/2015/294850

**Published:** 2015-08-09

**Authors:** S. B. Corvino, G. T. Volpato, M. V. C. Rudge, D. C. Damasceno

**Affiliations:** ^1^Laboratory of Experimental Research on Gynecology and Obstetrics, Gynecology, Obstetrics and Mastology Graduate Course, Botucatu Medical School, São Paulo State University (Unesp), 18618-970 Botucatu, SP, Brazil; ^2^Institute of Biological and Health Sciences, University Center of Araguaia, 78600-000 Barra do Garças, MT, Mato Grosso Federal University (UFMT), Brazil

## Abstract

This study aimed at evaluating the effect of swimming before and during pregnancy on rats born with intrauterine growth restriction (IUGR) and their offspring. For this, nondiabetic and streptozotocin-induced severely diabetic (SD) pregnant rats were mated and generated offspring with appropriate (control, C) and small (IUGR) for pregnancy age, respectively. Following that, C and IUGR groups were further distributed into nonexercised control (C), exercised control (Cex), nonexercised IUGR (IUGR), and exercised IUGR (IUGRex). IUGR rats presented lower mating rate than control rats. Regardless of physical exercise IUGR rats presented decreased body weight from birth to lactation. At 90 days of life, IUGR rats presented glucose intolerance. Maternal organ weights were increased and relative adiposity of IUGRex rats was lower than Cex. IUGR and IUGRex offspring presented reduced body weight than C and Cex, respectively. IUGRex dams presented an increased rate of appropriate for pregnancy age newborns. IUGEex male and female offspring relative brain weight was increased compared with Cex. Therefore, swimming before and during pregnancy prevented glucose intolerance, reduced general adiposity, and increased maternal and offspring organ weight in rats, showing the benefit of physical exercise for IUGR rats.

## 1. Introduction

Maternal body composition has important effects on the offspring. Extremes of maternal body composition in pregnancy are associated with adverse long-term offspring outcomes [1]. Several clinical and experimental studies show that suboptimal uterine and early neonatal life environments alter development and predispose the individual to lifelong health problems. The Developmental Origins of Health and Disease (DOHaD) has become well estabilished over the years, with animal studies reinforcing the outcomes of nutrient restriction and overfeeding during pregnancy [2]. Therefore, fetal programming is extremely important and involves many diseases that can have an impact on successive generations [3–5].

Experimental models using laboratory animals are relevant to expand and improve the understanding of the pathophysiological mechanisms involved in an inappropriate intrauterine environment. Therefore, it is necessary to develop adequate experimental models [6]. Several experimental models to generate an inadequate maternal environment are avaiable, including corticosteroids [7], decreased uterine blood flow through the uterine arteries bilaterally ligament [7–13], chronic hypertension [14], protein malnutrition [5, 15], and uncontrolled type 1* Diabetes Mellitus* [16–20].

Maternal hyperglycemia during pregnancy causes complications for both mother and offspring. Previous studies showed that adult female rats with chemically induced diabetes by streptozotocin (STZ) presented high glycemia (>300 mg/dL: severe diabetes) and their offspring were born small for pregnancy age (SPA) due to intrauterine growth restriction (IUGR) [19, 20], which can be due to maternal hyperglycemia leading to fetal hyperglycemia, causing pancreatic beta cell exhaustion and consequently hypoinsulinemia [21, 22].

Several procedures are employed to control maternal glycemia and prevent embryofetal development impairment. Traditionally, the most used therapeutic resource to control blood glucose is the association between diet and insulin. However, the efficacy of alternative aprroaches, such as exercise, is being tested [23, 24]. In general, the benefits of regular physical activity are improvement of cardiac performance, reduction of body fat index and water retention, better glycemic control, and better perinatal outcome [25]. Physical activity has been known for its role in controlling glycemic levels by direct or indirect effects on insulin action [26]. However, a major question remains regarding the correlation between the potential benefits and risks of physical exercise on fetal development during human pregnancy [27, 28]. A previous study performed in our laboratory demonstrated that swimming applied to diabetic rats from day 7 (after embryo implantation) to day 20 of pregnancy led to an improvement in maternal lipid metabolism, showing beneficial results. Besides, these rats presented reduced embryonic death rates (resorption) compared with diabetic nonexercised dams. However, these rats showed fetuses presenting small weight for pregnancy age [19, 29]. Another study performed by Corvino et al. (2015) [30] showed that streptozotocin-induced diabetic adult female rats (severe diabetes) presented intrauterine growth restricted offspring (IUGR). These adult IUGR rats were submitted to a swimming program during pregnancy similar to that of Volpato et al. 2009 [19], 2006 [29]. Corvino and colleagues' findings [30] showed that at day 10 postpartum, maternal weight gain and blood glucose level were unchanged. Besides, there was improved maternal lipid profile and increased insulin sensitivity, showing the beneficial results of this type of exercise for the maternal organism. However, it was verified that the offspring of IUGR rats submmited to the swimming program were small for pregnancy age, suggesting intrauterine growth restriction. This result suggests that intensity, type, and period of swimming may interfere with embryofetal development [30].

Considering the negative results obtained by Volpato et al. [29] in the offspring of IUGR rats and given the results from Vega et al. [31], who applied a different model of exercise (reduced duration and fewer times a week) to improve maternal metabolism and perinatal outcomes, we hypothesized that the development of a new swimming model applied to IUGR rat in adulthood could improve intrauterine environment and promote fetal programming in the offspring, thus preventing the appearance of diseases in adulthood. Therefore, the aim of the present study was to evaluate the effect of swimming before and during pregnancy on rats born with intrauterine growth restriction (IUGR) and their offspring.

## 2. Materials and Method

### 2.1. Animals

Female and male Wistar rats (CEMIB, UNICAMP, Campinas, São Paulo State, Brazil) weighing approximately 200 grams (g) were housed in a certified animal care. Food and water were provided* ad libitum*. The rats were maintained in Laboratory of Experimental Research on Gynecology and Obstetrics under controlled conditions (temperature 22 ± 2°C, humidity 55 ± 5%, and 12 h light/dark cycle).

### 2.2. Diabetes Induction: To Create an Uncontrolled Intrauterine Environment for Obtaining Intrauterine Growth Restricted (IUGR) Offspring

#### 2.2.1. Severe Diabetes Induction

Severe diabetes was induced at adult life of female rats (approximately at 90 days of age) by beta cytotoxic drug (Streptozotocin, STZ; Sigma Chem. Company, USA). STZ was dissolved in a citrate buffer (0.1 mol/L, pH 4.5) and intravenously (i.v.) administered at a dose of 40 mg/kg body weight [18–20]. Control rats received only citrate buffer using similar route and administration period. Seven days after STZ injection, the diabetic state was confirmed by blood glucose levels ≥ 300 mg/dL using a conventional glucometer (OneTouch Ultra—Johnson & Johnson). For nondiabetic rats, the inclusion criteria used was blood glucose levels ≤ 120 mg/dL. Glycemic values were expressed in milligrams per deciliter (mg/dL). After one week of diabetes confirmation or buffer administration (control), all adult female rats were mated overnight with nondiabetic adult male rats. The morning on which spermatozoa were found in the vaginal smear was designated pregnancy day 0 [19]. The offspring was born by spontaneous delivery.

#### 2.2.2. Sexing and Body Weight Classification for Offspring

After vaginal delivery, all newborns (NB) were examined for sex determination by the anogenital distance, which is about twice larger in the male than in the female [32]. Following that, the female offspring were separated and classified by the mean ± 1.0 × standard deviation (SD) according to the mean values of fetal weights of the control group as small for pregnancy age (SPA) when weight was smaller than mean of the control group −1.0 × SD; appropriate for pregnancy age when weight was into of the mean values of control group (mean ± 1.0 × SD); and large for pregnancy age (LPA) when weight was superior to mean of control group + 1.0 × SD [33]. The data were presented as percentual values. The female newborns born to nondiabetic dams and classified as appropriate for pregnancy age (APA = 93.2%) were included and denominated as control group, and the female offspring born to severe diabetic dams and classified as small for pregnancy age (SPA = 70.9%) were included and named as intrauterine growth restriction group (IUGR) [33]. After fetal classification, only eight newborns (rather female) were maintained with their dams for lactation up to weaning period (day 21 postnatal). Following that, these offspring after weaning were maintained until adulthood (approximately 90 days of life). All nondiabetic and diabetic rats dams were anesthetized, killed, and discarded during the experiment.

### 2.3. Body Weight, Oral Glucose Tolerance Test (OGTT), and Biochemical Determinations before Pregnancy of Control (C) and Intrauterine Growth Restricted (IUGR) Dams

At mornings of days 90 and 120 of life, the maternal body weights were recorded and oral glucose tolerance test (OGTT) was performed. For OGTT, after fasting for 6 hours, glycemia was verified (timepoint 0); then a glucose solution (200 g/L) was administered by* gavage* at a final dose of 2 g/kg body weight. Following that, the blood samples were obtained from a cut tip tail for glycemic determinations using a specific glucosemeter at 30, 60, and 120 minutes (min) [34].

### 2.4. Experimental Groups

For distribution of control and IUGR rats, submitted or not to swimming, the experimental groups were denominated:

- *C* (*control*): nonexercised APA female rats;
- *Cex* (*exercised control*): APA female rats exercised prior to and during pregnancy;
- *IUGR* (*intratuerine growth restriction*): nonexercised SPA female rats;
- *IUGRex* (*intratuerine growth restriction*): SPA female rats exercised prior to and during pregnancy.

### 2.5. Physical Exercise (Swimming Program) of Control (C) and Intrauterine Growth Restricted (IUGR) Rats: Prior to and during Pregnancy

At day 90 of life, one month before the mating period, C and IUGR rats were randomly selected to begin swimming program modified of other two exercise protocols: as the swimming Volpato et al. (2006) [29] and similar to the exercise time of Vega et al. (2013) [31], who used the wheel system for the rats. The Cex and IUGRex rats (Generation F1) were exposed to swimming program three times per week in a cage (100 × 70 × 60 cm) containing water at a depth of 40 cm (sufficient for them to be encouraged to swim) at 32 ± 2°C, and without additional overhead to the body during 15 min, followed by 15 min rest and a second 15 min swimming between 9 AM and 10 AM. Throughout the study, the rats were submitted to the swimming program during three times a week.

### 2.6. Obtaining Pregnant Rats

At day 120 of life, all groups (control, C; C exercised, Cex; IUGR and IUGR exercised, IUGRex) of rats were submitted for mating using similar proceedings to mother rats. During pregnancy, the pregnant rats were maintained in the individual cages. The exercised groups (Cex and IUGRex) continued swimming program during this period until day 20 of pregnancy.

### 2.7. Body Weight, OGTT, and Glycemic Determinations during Pregnancy of Control (C), Exercised Control (Cex), IUGR, and Exercised IUGR (IUGRex) Dams

At mornings of days zero (early pregnancy), 7 (embryonic period), 14 (fetal period), and 20 of pregnancy (end of pregnancy, at term pregnancy), the maternal body weights and postprandial glycemia were determined for evaluation of swimming effect. All blood samples were obtained by venous puncture of the tail. Blood glucose concentrations were measured by conventional glucometer and these values were expressed in mg/dL.

At the day 17 of pregenacy, OGTT was performed in all these rats to evaluate the glucose tolerance following the methodology described in Section 2.3.

### 2.8. Body Weight, OGTT, and Glycemic Determinations after Delivery of Control (C), Exercised Control (Cex), IUGR, and Exercised IUGR (IUGRex) Dams and Their Newborns

On day 1 after vaginal delivery, the mother rats and their offspring were weighed. Then, the sexing and body weight classification of these newborns were performed as described in Section 2.2.2 of this experiment.

At days 5 and 9 of lactation, the dams and their offspring were again weighed and blood samples were obtained by venous puncture of the tail for blood glucose concentrations were measured by conventional glucometer (mg/dL).

At day 10 after delivery, the dams and their offspring were weighed and anesthetized with sodium pentobarbital (Hypnol, 50 mg/kg body weight). The maternal and newborn heart, lung, pancreas, liver, adipose tissues (peritoritoneal, periovariane, periuterine, pancreatic, and sternal; only mothers) were collected. These organs were dissected and weighed to obtain the relative weight (absolute weight/body weight × 100). Regarding adipose tissues, the calculation of total fat (sum of all adipose tissues) and relative weight (total body fat/body weight × 100).

### 2.9. Measurement of Maternal Milk Production of Control (C), Exercised Control (Cex), IUGR, and Exercised IUGR (IUGRex) Dams

At 7:00 AM at days 5 and 9 of lactation, pups were removed from the mothers for 4 h and their dams ate* ad libitum*. After that, these dams were weighed at the beginning (T1) and end (T2) of the 4 h period. The indirect calculation of milk production (g) was the weight (T1) and weight (T2) [35].

### 2.10. Statistical Analyses

The data were presented as mean ± standard deviation. To avoid overinfluence of data from a single mother in study, the females for each group came from different litters. Respecting that the homogeneity among experimental units is one of the basics of experimental design and considering that SPA and APA are biologically different organisms, the comparison between sedentary SPA versus sedentary APA and exercited SPA versus exercited was performed. Student's unpaired *t*-test for normal distribution and Mann-Whitney for abnormal distribution of data were used to compare only two groups. The proportion data were analyzed by Fisher's exact test. The oral glucose tolerance test was analyzed by Gamma distribution followed by repeated measures. SAS software (version 9.3) was applied for all statistical analyses. *p* < 0.05 was considered as statistical significance limit.

### 2.11. Ethical Aspects

The Ethics Committee on Animal Experiments of the Botucatu Medical School,UNESP, approved all experimental procedures performed in this study (Protocol number: 938/2012).

## 3. Results

### 3.1. Maternal Data

#### 3.1.1. Oral Glucose Tolerance Test (OGTT)

**Figure 1:**
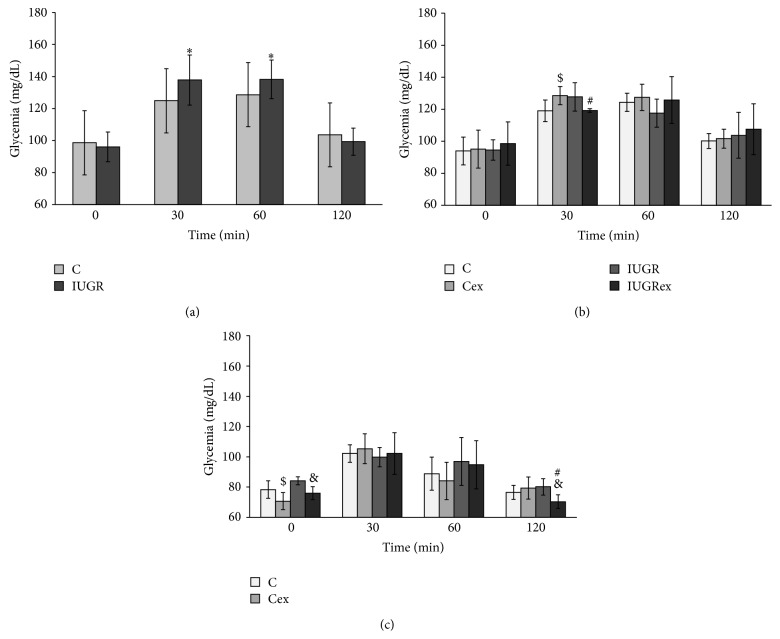
Oral glucose tolerance test at day 90 of life (a), at day 120 of life (b), and at day 17 of pregnancy (c) of control not exercised (C, *n* = 6), exercised control (Cex, *n* = 8), intrauterine growth restricted not exercised (IUGR, *n* = 5), and exercised IUGR (IUGRex, *n* = 5) dams. Values are expressed as mean ± standard deviation. ^$^ *p* < 0.05: statistically significant difference between C and Cex. ^*∗*^ *p* < 0.05: statistically significant difference between C and IUGR. ^#^ *p* < 0.05: statistically significant difference between Cex and IUGRex. ^&^ *p* < 0.05: statistically significant difference between IUGR and IUGRex. (Gamma distribution followed by repeated measures.)

At 90 days old, it was oberved that the blood glucose levels were increased in the timepoints 30 and 60 minutes (min) in the IUGR group (137.80 ± 15.63 and 138.20 ± 12.04 mg/dL, resp.) compared to those of control group (124.86 ± 6.71 and 127.64 ± 6.33 mg/dL, resp.) (Figure 1(a)). With 120 days of age, there was increased glycemia in the Cex group (95.12 ± 11.88 mg/dL) compared to control group (C) (94.00 ± 8.64 mg/dL) and decreased blood glucose levels in IUGRex group (119.26 ± 1.09 mg/dL) compared to Cex group (128.62 ± 5.68 mg/dL) only in timepoint 30 min (Figure 1(b)). On day 17 of pregnancy, at the beginning of OGTT (timepoint zero before the glucose overload), the Cex (70.75 ± 5.65 mg/dL) and IUGRex (76.00 ± 4.24 mg/dL) groups presented reduced blood glucose levels compared to their respective control groups (C = 78.33 ± 5.78 and IUGR = 84.20 ± 2.68), respectively. At the end of OGTT (timepoint 120 min), the IUGRex group (70.40 ± 4.56) presented reduced glycemia in relation to Cex (79.37 ± 7.33) and IUGR (80.20 ± 5.36) groups (Figure 1(c)).

#### 3.1.2. Body Weight

**Figure 2:**
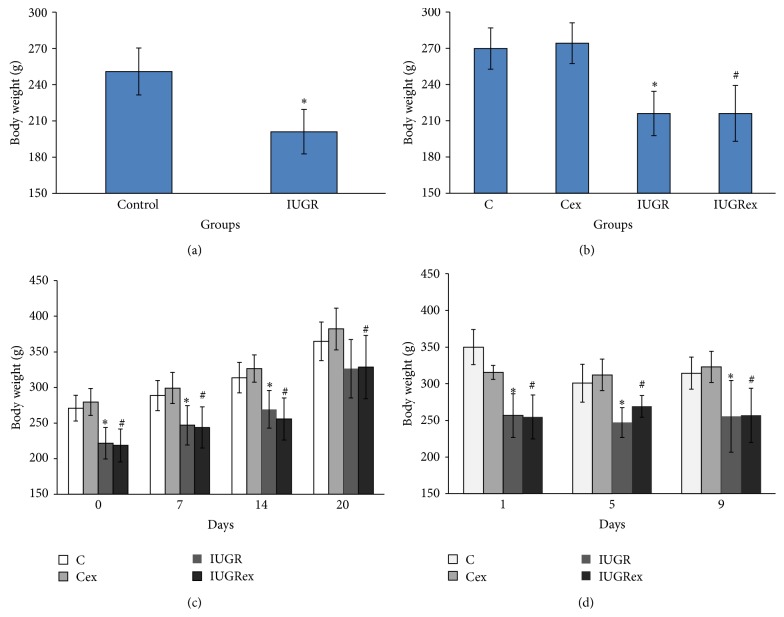
Body weight at day 90 (a), at day 120 of life (b), during pregnancy (c), and during lactation (d) of control not exercised (C, *n* = 6), exercised control (Cex, *n* = 8), intrauterine growth restricted not exercised (IUGR, *n* = 5), and exercised IUGR (IUGRex, *n* = 5) dams. Values are expressed as mean ± standard deviation. ^*∗*^ *p* < 0.05: statistically significant difference between C and IUGR (*t* test). ^#^ *p* < 0.05: statistically significant difference between Cex and IUGRex (*t* test).

Figure 2 shows the evolution of body weight. With 90 days of age, the rats of the IUGR group presented lower body weights compared to those of control group (Figure 2(a)). On the day 120 of life, the IUGR and IUGRex groups also showed reduced weight (*p* < 0.05) compared to their respective control groups (C and Cex) (Figure 2(b)). During pregnancy, there was an increase of maternal body weight in all groups, but IUGRex and IUGR groups on days 0, 7, and 14 of pregnancy had lower body weights in relation to their respective C and IUGR groups, respectively. The IUGRex rats also showed decreased weight on the day 20 of pregnancy compared to Cex group (Figure 2(c)). During the lactation period (days 1, 5, and 9), the IUGR IUGRex groups showed decreased body weights compared to C and Cex groups, respectively (Figure 2(d)).

#### 3.1.3. Litter Size

It was verified that there was no change regarding the litter size and the number of newborns among experimental groups (C = 9.80; Cex = 11.37; IUGR = 10.80 and IUGRex = 11.25 newborns).

#### 3.1.4. Milk Production

The experimental groups showed a milk mean production on day 5 of lactation in the control group (3.33 ± 1.12 g); Cex (4.93 ± 3.12 g); IUGR (4.40 ± 0.80 g) and IUGRex (3.91 ± 12.02 g) and on day 9 in the control group (3.83 ± 3.18 g); Cex (2.50 ± 7.20 g); IUGR (3.90 ± 3.92 g) and IUGRex (5.48 ± 2.55 g). There was no statistically significant difference in milk production among experimental groups.

#### 3.1.5. Reproductive Outcomes

In relation to parental generation, 64 female rats were injected with streptozotocin and of these 100% presented blood glucose concentration above 300 mg/dL. In the nondiabetic group, 7 rats received citrate buffer and 100% of them presented glycemia below 120 mg/dL. At adult life, all nondiabetic female rats and 27 diabetic female rats mated. Of these, all nondiabetic rats (100%) and 13 severe diabetic rats reached at term pregnancy. The severe diabetic dams presented lower alive fetuses compared to nondiabetic rats (Table 1).

#### 3.1.6. Organ and Adipose Tissues Relative Weights

The relative weight of heart of Cex rats was reduced compared to C group. In IUGRex group, there is an increase compared to Cex group (*p* < 0.05). The relative weight of pancreas and lung was increased in IUGRex group compared to the group Cex. There was no statistically significant difference in the relation to relative weights of liver and mammary gland among the different experimental groups (*p* > 0.05) (Table 2).

The relative weight of the adipose tissues (pancreatic, peritoneal, periovariane, and sternal) was reduced in IUGRex rats compared to Cex rats. Regarding peritoneal and periovariane adipose tissues, it was verified that there was a reduction in relative weight in IUGRex rats compared to the IUGR group. The relative weight of sternal adipose tissue was also reduced in IUGR group compared to the C group (Table 2).

#### 3.1.7. Total and Relative Adiposity/Fat

In relation to total fat, the IUGR group showed a decrease compared to control group (C) and the IUGRex group also showed reduced adiposity compared to Cex and IUGR groups. The relative fat was reduced in IUGRex rats in relation to Cex and IUGR rats (Table 2).

### 3.2. Newborn Data 

#### 3.2.1. Body Weight

**Figure 3:**
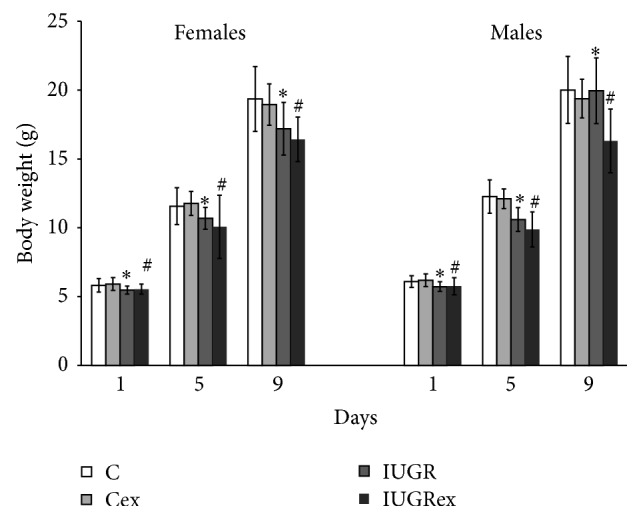
Body weight of females from control not exercised (C, *n* = 21), exercised control (Cex, *n* = 33), intrauterine growth restricted not exercised (IUGR, *n* = 21), and exercised IUGR (IUGRex, *n* = 22). Body weight of males from control not exercised (C, *n* = 22), exercised control (Cex, *n* = 30), intrauterine growth restricted not exercised (IUGR, *n* = 19), and exercised IUGR (IUGRex, *n* = 18). Values are expressed as mean ± SD. ^*∗*^ *p* < 0.05: statistically significant difference between C and IUGR. ^#^ *p* < 0.05: statistically significant difference between Cex and IUGRex. (*t* test for data at days 1 and 9 of life and Mann-Whitney Test for data at day 5 of females; *t* test for data at days 1 and 5 of life and Mann-Whitney Test for data at day 9 of males).

The females and males newborns showed reduced body weights (days 1, 5, and 9 of postnatal life) in IUGR and IUGRex groups in relation to C and Cex groups, respectively (Figure 3).

#### 3.2.2. Blood Glucose Levels

The glycemic mean of female newborns at day 5 (C group = 117.65 ± 9.20 mg/dL; Cex group = 113.84 ± 10.45 mg/dL; IUGR group = 114.50 ± 10.59 and IUGRex groups = 117.43 ± 10.30) and day 9 (C group = 119.00 ± 21.64 mg/dL; Cex group = 117.52 ± 13.57 mg/dL; IUGR group = 113.69 ± 8.81 and IUGRex groups = 107.61 ± 13.32) presented no difference compared to days 5 and 9. The glycemic mean of male newborns at day 5 (C group = 110.71 ± 8.52 mg/dL; Cex group = 115.93 ± 11.01 mg/dL; IUGR group = 108.16 ± 14.13 and IUGRex groups = 116.65 ± 12.65) and day 9 (C group = 114.00 ± 12.12 mg/dL; Cex group = 114.58 ± 12.60 mg/dL; IUGR group = 104.82 ± 13.59 and IUGRex groups = 109.93 ± 17.61) presented also no difference compared to days 5 and 9, regardless of the groups where mothers were inserted.

The maternal blood glucose levels were not correlated (*p* > 0.05) with the blood glucose levels of their newborns (data not shown).

#### 3.2.3. Organ Relative Weights

The newborn females and males showed that relative weights of brain and lung increased in IUGR and IUGRex groups compared to C and Cex groups, respectively. Only female newborns showed increased relative weight of the lung in Cex group compared to the control group (C). The male offspring showed an increase in heart weight in IUGRex group compared to those of Cex rats (Table 3).

## 4. Discussion

In the present study, the rats born with intrauterine growth restriction (IUGR) presented glucose intolerance at adulthood. These findings corroborate previous study of our laboratory, where diabetic rats presented two or more timepoints superior to 140 mg/dL during OGTT at day 90 of life [36].

After 30 and 50 days of swimming application, the rats of IUGRex group showed reduced blood glucose levels in OGTT at day 120 of life and 17 of pregnancy, respectively, showing that exercise was beneficial for these rats. Physical exercise and insulin physiologically stimulate glucose transport in skeletal muscle [37, 38]. Exercise positively helps in fetal weight and morphological development of the organs of fetuses, prevents* Diabetes Mellitus* onset, and regulates lipid metabolism [26, 29]. These experimental data are compatible findings of Dallaqua et al. [39]. Another study that corroborates our results is a model of uteroplacental insufficiency in rats showing that the first generation (F0) originated restricted female newborns (F1). On day 18 of pregnancy, there was no reduction in plasma glucose of the same manner compared to that of control group during the OGTT, indicating a glucose intolerance in all timepoints of this test [11]. In our study, the glucose intolerance status was reverted by swimming program.

In the present experiment, regardless of practicing swimming, the rats of IUGR group were born and remained with lower weights throughout the experiment (days 90 and 120 of life, pregnancy, and lactation period), showing how these rats showed no growth. The catch-up growth can be defined as a realignment of individual genetic growth potential after intrauterine growth retardation (IUGR) [40]. According to Holemans et al. [22, 41], IUGR rats obtained from the diabetes induction in rats on the 11th day of pregnancy presented lower body weight during their entire postnatal life, corroborating our findings. Furthermore, it was found that an uteroplacental insufficiency in F0 females led to a reduced body weight of F1 female newborns at postnatal day 1 and these restricted females remained with lower weights at all ages studied [11–13]. Contradictorily, other authors found increased body weight, featuring catch-up growth on the model of uteroplacental insufficiency in F0 females. These adult rats had newborns with low weights at postnatal day 1, but caught up similarly to control rats at 4 months [10, 42].

The absence of catch-up growth may play important role in protecting them from adverse metabolic outcomes in the long term and to prevent the deterioration of in vivo insulin action that occurs with age and, as a result, glucose levels are more easily maintained [12], corroborating our results related to glycemia during pregnancy and lactation periods. Besides, the catch-up growth in IUGR might differently influence the type 2 diabetes pathogenesis. The insulin resistance might play a major role in the subjects who show catch-up growth while insulin secretion defect or impaired *β*-cell development plays a major role in the subjects who fail to undergo catch-up growth. Corvino et al. [30] also verified no catch-up growth in IUGR rats, suggesting that the IUGR groups presented defects in insulin action that precedes insulin secretion impairment, leading to insulin resistance development. However, the swimming program apllied to IUGR pregnant rats improved insulin sensitivity.

In our study, the rats born with IUGR who practiced swimming presented weight gain during pregnancy and reduced total and relative adiposity, showing beneficial effect of exercise for these rats. This could prevent obesity onset, which develops in adulthood of offspring born of IUGR [43, 44]. However, another study using voluntary exercise in wheel before and during pregnancy of obese rats (MO) observed that these mothers gained less weight during pregnancy without changes in offspring weight at birth; MEx (exercised obese rats) improved maternal carbohydrate metabolism but the signs of dysfunctional carbohydrate metabolism remained, suggesting that the level of exercise, beginning even before pregnancy, could not completely suppress the effects of MO and importantly, short periods (30 min a day) of exercise for 1 month clearly provide benefits to both mother and offspring [31]. Care is necessary drawing conclusions from this interesting finding as exercise may have different effects on different fat depots. We would pose the testable hypothesis that exercise-induced mechanisms change some adipocyte metabolic pathways leaving others. A different exercise regimen may be required to modify them [31]. In contrast, regular exercise is known to reduce body fat [45], with the majority of research focused on exercise-induced fatty acid oxidation [46]. Besides, these results reinforce the importance of the type and intensity of exercise as well as the duration and frequency of exercise sessions to carefully balance between potential benefits and potential harmful effects. Additional attention should be given to progression in intensity over time.

Regarding the relative weights of maternal organs, our results showed that the relative weights of heart, pancreas, and lung of exercised IUGR rats were similar to the control group. In a model of uteroplacental insufficiency in rats, it was observed that these dams generated restricted females newborns and it was verified that organ relative weights (heart and pancreas) were also not different between control and restricted pregnant groups at day 19 of pregnancy [10].

In this study, it was found no changes in litter size. Similarly, it was demonstrated that the total (male and female) F2 litter size was not different between control and restricted rats in others investigations [11, 13].

In relation to F2 male and female newborns from mothers restricted (IUGR), regardless of the swimming practice, there was a higher percentage of newborns classified as adequate for pregnancy age (data not shown), showing that the exercise was not harmful for offspring growth of rats. In contrast with the results of Damasceno et al., 2013 [47], and Volpato et al., 2015 [48], who showed that swimming (60 min/day, 6 times/week) in nondiabetic rats led to an increased rate of newborns classified as small for age of pregnancy, confirming the intrauterine growth restriction. Our results indicate that the unfavorable intrauterine environment is modified positively by the practice of maternal exercise.

The glycemia of female and male newborn showed no changes in the perinatal period. It has been shown in our study that the male and female newborns of IUGRex mothers group showed an increase in the relative weight of the heart, brain, and lung and increased relative weight of brain and lung (females and males) in the IUGR mothers group was also observed. Brain sparing is a feature of intrauterine growth retardation (IUGR), which implies that there is a redistribution of metabolic supply so that body growth slows to a greater extent than brain growth [49]. Our findings suggest that swimming program protected the brain of offspring born into an unadequate intrauterine environment.

In summary, there is ample evidence that an abnormal intrauterine environment can induce alterations in fetal metabolism with persisting consequences in late life and successive generations. Our data show that the newborns from diabetic rats were born with IUGR and developed glucose intolerance at adulthood. However, when these rats were subjected to a swimming program before and during pregnancy, the intolerance glucose was prevented. Besides, there was a reduced adiposity general preventing the possibilities of developing an obesity status, an increased organ weight in maternal organism and in offspring, and increased rate of newborn classified as adequate for pregnancy age, showing the beneficial effect of physical exercise in IUGR rats throughout two successive generations.

## 5. Perspective

Studies to translate findings of animal models to human practice are found in the literature but the transgenerational studies using adult intrauterine growth restricted rats are rare, especially considering pregnant and submitting to physical exercise. Then this investigation was performed with adult intrauterine growth restricted rats submitted to swimming program before and during pregnancy to evaluate the exercise effect on maternal organisms and their offspring. Our findings showed that the swimming program prevented glucose intolerance of adult intrauterine growth restricted rats and obesity and favored an increase in organs and body weight of their offspring, suggesting beneficial effect of swimming program in two rat successive generations.

## Figures and Tables

**Table 1:** Outcomes of female rats from parental and first generation.

	Parental generation			
	Nondiabetic		Severe diabetic	
Number of rats	7		64^*∗*^	
Number of mated female rats	7/7 (100%)		27/64 (42.24%)^*∗*^	
Number of rats with at term pregnancy	7/7 (100%)		13/27 (48.10%)^*∗*^	
Littler size/rat	13.14		7.84^*∗*^	

	First generation			
	APA/Control (from nondiabetic dam)		SPA/IUGR (from diabetic dam)	

Female offspring at birth	29		37	
Number of alive female offspring at 3 months	23/29 (79.31%)		18/37 (51.35%)	
Number of mated female rats	15/23 (65.21%)		10/18 (55.55%)	

	C	Cex	IUGR	IUGRex

Number of rats with at term pregnancy	6	8	5	5

^*∗*^ *p* < 0.05: compared to nondiabetic/control groups (Fisher's exact test).

**Table 2:** Maternal relative weight of rats (%) and weight of different adipose tissues (%) at day 10 of lactation of control not exercised (C), exercised control (Cex), intrauterine growth restricted not exercised (IUGR), and exercised IUGR (IUGRex) dams.

Organs	Groups			
	C (*n* = 6)	Cex (*n* = 8)	IUGR (*n* = 5)	IUGRex (*n* = 5)
Heart (%)^a^	0.35 ± 0.04	0.28 ± 0.01^$^	0.33 ± 0.04	0.34 ± 0.01^#^
Pancreas (%)^b^	0.24 ± 0.03	0.22 ± 0.03	0.30 ± 0.05	0.25 ± 0.01^#^
Lung (%)^a^	0.50 ± 0.06	0.44 ± 0.08	0.51 ± 00.15	0.62 ± 0.11^#^
Liver (%)^b^	3.45 ± 0.121	3.47 ± 0.19	3.42 ± 0.58	3.78 ± 0.48
Adipose tissues				
Peritoneal (%)^b^	1.34 ± 0.59	1.51 ± 0.265	0.92 ± 0.33	0.42 ± 0.10^#&^
Periovarian (%)^b^	0.28 ± 0.12	0.36 ± 0.09	0.36 ± 0.18	0.15 ± 0.03^#&^
Periuterine (%)^a^	0.70 ± 0.22	0.60 ± 0.21	0.51 ± 0.25	0.41 ± 0.17
Pancreatic (%)^a^	0.19 ± 0.10	0.22 ± 0.66	0.21 ± 0.10	0.12 ± 0.03^#^
Sternal (%)^b^	0.07 ± 0.03	0.06 ± 0.02	0.02 ± 0.007^**∗**^	0.03 ± 0.007^#^
Total fat (g)^b^	8.41 ± 2.52	9.92 ± 4.11	5.15 ± 2.39^**∗**^	2.50 ± 1.39^#&^
Relative fat (%)^b^	0.85 ± 0.29	0.79 ± 0.15	0.67 ± 0.18	0.43 ± 0.08^#&^

Values are presented as percentual mean ± standard deviation.

^$^ *p* < 0.05: statistically significant difference between C and Cex.

^*∗*^ *p* < 0.05: statistically significant difference between C and IUGR.

^#^ *p* < 0.05: statistically significant difference between Cex and IUGRex.

^&^ *p* < 0.05: statistically significant difference between IUGR and IUGRex.

^a^ *t* test; ^b^Mann Whitney test.

**Table 3:** Relative weight of organs of newborns from control not exercised (C), exercised control (Cex), intrauterine growth restricted not exercised (IUGR), and exercised IUGR (IUGRex) dams at day 10 of lactation.

	Groups			
	C	Cex	IUGR	IUGRex
Females	*n* = 21	*n* = 33	*n* = 21	*n* = 22
Brain (%)^a^	3.61 ± 0.56	3.56 ± 0.57	4.09 ± 0.43^*∗*^	4.17 ± 0.46^#^
Pancreas (%)^a^	0.19 ± 0.05	0.19 ± 0.05	0.19 ± 0.05	0.18 ± 0.04
Lung (%)^b^	1.76 ± 0.09	1.94 ± 0.50^$^	1.98 ± 0.20^*∗*^	1.96 ± 0.13^#^
Heart (%)^a^	0.57 ± 0.07	0.54 ± 0.07	0.58 ± 0.10	0.54 ± 0.07
Liver (%)^a^	2.23 ± 0.26	2.30 ± 0.25	2.16 ± 0.34	2.28 ± 0.25
Males	*n* = 22	*n* = 30	*n* = 19	*n* = 18
Brain (%)^a^	3.15 ± 0.72	3.51 ± 0.76	4.13 ± 0.51^*∗*^	3.98 ± 0.55^#^
Pancreas (%)^b^	0.16 ± 0.04	0.19 ± 0.04	0.18 ± 0.08	0.19 ± 0.06
Lung (%)^b^	1.81 ± 0.16	1.83 ± 0.17	2.04 ± 0.22^*∗*^	2.04 ± 0.19^#^
Heart (%)^a^	0.52 ± 0.05	0.53 ± 0.06	0.56 ± 0.11	0.58 ± 0.08^#^
Liver (%)^a^	2.00 ± 0.75	2.32 ± 0.26	2.17 ± 0.35	2.30 ± 0.27

Values are presented as percentual mean ± standard deviation.

^$^ *p* < 0.05: statistically significant difference between C and Cex.

^*∗*^ *p* < 0.05: statistically significant difference between C and IUGR.

^#^ *p* < 0.05: statistically significant difference between Cex and IUGRex.

^a^ *t* test, ^b^Mann-Whitney test.

## References

[1] Godfrey K. M., Barker D. J. P. (2000). Fetal nutrition and adult disease. *The American Journal of Clinical Nutrition*.

[2] Nathanielsz P. W. (2006). Animal models that elucidate basic principles of the developmental origins of adult diseases. *ILAR Journal*.

[3] Fernandez-Twinn D. S., Ozanne S. E. (2006). Mechanisms by which poor early growth programs type-2 diabetes, obesity and the metabolic syndrome. *Physiology and Behavior*.

[4] Aerts L., Van Assche F. A. (2006). Animal evidence for the transgenerational development of diabetes mellitus. *International Journal of Biochemistry & Cell Biology*.

[5] Zambrano E. (2009). The transgenerational mechanisms in developmental programming of metabolic diseases. *Revista de Investigación Clínica*.

[6] López-Soldado I., Herrera E. (2003). Different diabetogenic response to moderate doses of streptozotocin in pregnant rats, and its long-term consequences in the offspring. *Experimental Diabesity Research*.

[7] Nyirenda M. J., Lindsay R. S., Kenyon C. J., Burchell A., Seckl J. R. (1998). Glucocorticoid exposure in late gestation permanently programs rat hepatic phosphoenolpyruvate carboxykinase and glucocorticoid receptor expression and causes glucose intolerance in adult offspring. *Journal of Clinical Investigation*.

[8] Jansson T., Lambert G. W. (1999). Effect of intrauterine growth restriction on blood pressure, glucose tolerance and sympathetic nervous system activity in the rat at 3-4 months of age. *Journal of Hypertension*.

[9] Simmons R. A., Templeton L. J., Gertz S. J. (2001). Intrauterine growth retardation leads to the development of Type 2 diabetes in the rat. *Diabetes*.

[10] Gallo L. A., Tran M., Moritz K. M. (2012). Cardio-renal and metabolic adaptations during pregnancy in female rats born small: implications for maternal health and second generation fetal growth. *Journal of Physiology*.

[11] Gallo L. A., Denton K. M., Moritz K. M. (2012). Long-term alteration in maternal blood pressure and renal function after pregnancy in normal and growth-restricted rats. *Hypertension*.

[12] Tran M., Gallo L. A., Wadley G. D., Jefferies A. J., Moritz K. M., Wlodek M. E. (2012). Effect of pregnancy for females born small on later life metabolic disease risk. *PLoS ONE*.

[13] Tran M., Gallo L. A., Jefferies A. J., Moritz K. M., Wlodek M. E. (2013). Transgenerational metabolic outcomes associated with uteroplacental insufficiency. *Journal of Endocrinology*.

[14] Bassan H., Bassan M., Pinhasov A. (2005). The pregnant spontaneously hypertensive rat as a model of asymmetric intrauterine growth retardation and neurodevelopmental delay. *Hypertension in Pregnancy*.

[15] Dahri S., Snoeck A., Reusens-Billen B., Remacle C., Hoet J. J. (1991). Islet function in offspring of mothers on low-protein diet during gestation. *Diabetes*.

[16] Damasceno D. C., Volpato G. T., de Mattos Paranhos Calderon I. (2002). Oxidative stress and diabetes in pregnant rats. *Animal Reproduction Science*.

[17] Damasceno D. C., Sinzato Y. K., Lima P. H. (2011). Effects of exposure to cigarette smoke prior to pregnancy in diabetic rats. *Diabetology and Metabolic Syndrome*.

[18] Volpato G. T., Calderon I. M. P., Sinzato S., Campos K. E., Rudge M. V. C., Damasceno D. C. (2011). Effect of *Morus nigra* aqueous extract treatment on the maternal-fetal outcome, oxidative stress status and lipid profile of streptozotocin-induced diabetic rats. *Journal of Ethnopharmacology*.

[19] Volpato G. T., Damasceno D. C., Kempinas W. G., Rudge M. V. C., Calderon I. M. P. (2009). Effect of exercise on the reproductive outcome and fetal development of diabetic rats. *Reproductive BioMedicine Online*.

[20] da Silva Soares de Souza M., Lima P. H. O., Sinzato Y. K., Rudge M. V. C., Pereira O. C. M., Damasceno D. C. (2009). Effects of cigarette smoke exposure on pregnancy outcome and offspring of diabetic rats. *Reproductive BioMedicine Online*.

[21] Pedersen J. (1954). Weight and length at birth of infants of diabetic mothers. *Acta Endocrinologica*.

[22] Holemans K., Aerts L., Van Assche F. A. (2003). Fetal growth restriction and consequences for the offspring in animal models. *Journal of the Society for Gynecologic Investigation*.

[23] Devlin J. T. (1992). Effects of exercise on insulin sensitivity in humans. *Diabetes Care*.

[24] Kim C. (2010). Gestational diabetes: risks, management, and treatment options. *International Journal of Women's Health*.

[25] Melzer K., Schutz Y., Boulvain M., Kayser B. (2010). Physical activity and pregnancy: cardiovascular adaptations, recommendations and pregnancy outcomes. *Sports Medicine*.

[26] Uriu-Hare J. Y., Keen C. L., Applegate E. A., Stern J. S. (1989). The influence of moderate exercise in diabetic and normal pregnancy on material and fetal outcome in the rat. *Life Sciences*.

[27] Zaidise I., Artal R., Bessman S. P., Artal R., Wiswell R. A., Drinkwater B. L. (1999). Metabolismo de combustíveis na gravidez—aspectos teóricos. *O Exercício na Gravidez*.

[28] Bessinger R. C., McMurray R. G. (2003). Substrate utilization and hormonal responses to exercise in pregnancy. *Clinical Obstetrics and Gynecology*.

[29] Volpato G. T., Damasceno D. C., Campos K. E., Rocha R., Rudge M. V., Calderon I. d. (2006). Avaliação do efeito do exercício físico no metabolismo de ratas diabéticas prenhes. *Revista Brasileira de Medicina do Esporte*.

[30] Corvino S. B., Netto A. O., Sinzato Y. K. (2015). Intrauterine growth restricted rats exercised at pregnancy: maternal-fetal repercussions. *Reproductive Sciences*.

[31] Vega C. C., Reyes-Castro L. A., Bautista C. J., Larrea F., Nathanielsz P. W., Zambrano E. (2015). Exercise in obese female rats has beneficial effects on maternal and male and female offspring metabolism. *International Journal of Obesity*.

[32] Damasceno D. C., Kempinas W. G., Volpato G. T. (2008). *Anomalias congênitas—Estudos experimentais*.

[33] Corvino S. B., Netto A. O., Macedo N. C. D., Calderon I. M. P., da Cunha Rudge M. V., Damasceno D. C. (2013). Repercussões do diabete de intensidade grave no crescimento corporal em sucessivas gerações de ratas. *Nutrição Brasil*.

[34] Santos T. M., Sinzato Y. K., Gallego F. Q. (2015). Extracellular HSP70 levels in diabetic environment in rats. *Cell Stress & Chaperones*.

[35] Bautista C. J., Boeck L., Larrea F., Nathanielsz P. W., Zambrano E. (2008). Effects of a maternal low protein isocaloric diet on milk leptin and progeny serum leptin concentration and appetitive behavior in the first 21 days of neonatal life in the rat. *Pediatric Research*.

[36] Gallego F. Q., Sinzato Y. K., Dallaqua B. (2015). Morphological analysis of pancreatic beta cells of diabetic female rats at different ages of life. *Palcenta*.

[37] Hayashi T., Wojtaszewski J. F. P., Goodyear L. J. (1997). Exercise regulation of glucose transport in skeletal muscle. *The American Journal of Physiology—Endocrinology and Metabolism*.

[38] Goodyear L. J., Kahn B. B. (1998). Exercise, glucose transport, and insulin sensitivity. *Annual Review of Medicine*.

[39] Dallaqua B., Saito F. H., Rodrigues T. (2012). Treatment with *Azadirachta indica* in diabetic pregnant rats: negative effects on maternal outcome. *Journal of Ethnopharmacology*.

[40] Simmons R. (2005). Developmental origins of adult metabolic disease: concepts and controversies. *Trends in Endocrinology & Metabolism*.

[41] Holemans K., Van Bree R., Verhaeghe J., Meurrens K., Van Assche F. A. (1997). Maternal semistarvation and streptozotocin-diabetes in rats have different effects on the in vivo glucose uptake by peripheral tissues in their female adult offspring. *Journal of Nutrition*.

[42] Mazzuca M. Q., Tare M., Parkington H. C., Dragomir N. M., Parry L. J., Wlodek M. E. (2012). Uteroplacental insufficiency programmes vascular dysfunction in non-pregnant rats: compensatory adaptations in pregnancy. *The Journal of Physiology*.

[43] Ravelli A. C. J., Van Der Meulen J. H. P., Osmond C., Barker D. J. P., Bleker O. P. (1999). Obesity at the age of 50 y in men and women exposed to famine prenatally. *American Journal of Clinical Nutrition*.

[44] Valdez R., Athens M. A., Thompson G. H., Bradshaw B. S., Stern M. P. (1994). Birthweight and adult health outcomes in a biethnic population in the USA. *Diabetologia*.

[45] Pate R. R., Pratt M., Blair S. N. (1995). Physical activity and public health: a recommendation from the centers for disease control and Prevention and the American College of Sports Medicine. *The Journal of the American Medical Association*.

[46] Karzel R. P., Friedman M. J., Artal R., Wiswell R. A., Drinkwater B. L. (1991). Orthopedic injuries in pregnancy. *Exercise in Pregnancy*.

[47] Damasceno D. C., Silva H. P., Vaz G. F. (2013). Diabetic rats exercised prior to and during pregnancy: maternal reproductive outcome, biochemical profile, and frequency of fetal anomalies. *Reproductive Sciences*.

[48] Volpato G. T., Damasceno D. C., Sinzato Y. K. (2015). Oxidative stress status and placental implications in diabetic rats undergoing swimming exercise after embryonic implantation. *Reproductive Sciences*.

[49] Lumbers E. R., Yu Z.-Y., Gibson K. J. (2001). The selfish brain and the barker hypothesis. *Clinical and Experimental Pharmacology and Physiology*.

